# Increased levels of XPA might be the basis of cisplatin resistance in germ cell tumours

**DOI:** 10.1186/s12885-019-6496-1

**Published:** 2020-01-06

**Authors:** Zuzana Cierna, Vera Miskovska, Jan Roska, Dana Jurkovicova, Lucia Borszekova Pulzova, Zuzana Sestakova, Lenka Hurbanova, Katarina Machalekova, Michal Chovanec, Katarina Rejlekova, Daniela Svetlovska, Katarina Kalavska, Karol Kajo, Pavel Babal, Jozef Mardiak, Thomas A. Ward, Michal Mego, Miroslav Chovanec

**Affiliations:** 1 Department of Pathology, Faculty of Medicine, Comenius University, Bratislava, Slovakia; 2 1st Department of Oncology, Faculty of Medicine, Comenius University, Bratislava, Slovakia; 3 St. Elisabeth Cancer Institute, Bratislava, Slovakia; 4 Department of Genetics, Cancer Research Institute, Biomedical Research Center, University Science Park for Biomedicine, Slovak Academy of Sciences, Bratislava, Slovakia; 5 2nd Department of Oncology, Faculty of Medicine, Comenius University, Bratislava, Slovakia; 6 National Cancer Institute, Bratislava, Slovakia; 7 Translational Research Unit, Comenius University, Bratislava, Slovakia; 8 Department of Molecular Oncology, Cancer Research Institute, Biomedical Research Center, University Science Park for Biomedicine, Slovak Academy of Sciences, Bratislava, Slovakia; 9 Faculty Hospital with Policlinics Skalica a.s., Skalica, Slovakia; 10 Department of Biomedical and Life Sciences, Faculty of Health and Medicine, Lancaster University, Lancaster, UK

**Keywords:** Germ cell tumours, DNA repair, Nucleotide excision repair, XPA, Prognostic marker

## Abstract

**Background:**

Germ cell tumours (GCTs) represent a highly curable malignity as they respond well to cisplatin (CDDP)-based chemotherapy. Nevertheless, a small proportion of GCT patients relapse or do not respond to therapy. As this might be caused by an increased capacity to repair CDDP-induced DNA damage, identification of DNA repair biomarkers predicting inadequate or aberrant response to CDDP, and thus poor prognosis for GCT patients, poses a challenge. The objective of this study is to examine the expression levels of the key nucleotide excision repair (NER) factors, XPA, ERCC1 and XPF, in GCT patients and cell lines.

**Methods:**

Two hundred seven GCT patients’ specimens with sufficient follow-up clinical-pathological data and pairwise combinations of CDDP-resistant and -sensitive GCT cell lines were included. Immunohistochemistry was used to detect the ERCC1, XPF and XPA protein expression levels in GCT patients’ specimen and Western blot and qRT-PCR examined the protein and mRNA expression levels in GCT cell lines.

**Results:**

GCT patients with low *XPA* expression had significantly better overall survival than patients with high expression (hazard ratio = 0.38, 95% confidence interval: 0.12–1.23, *p* = 0.0228). In addition, *XPA* expression was increased in the non-seminomatous histological subtype, IGCCCG poor prognosis group, increasing S stage, as well as the presence of lung, liver and non-pulmonary visceral metastases. Importantly, a correlation between inadequate or aberrant CDDP response and *XPA* expression found in GCT patients was also seen in GCT cell lines.

**Conclusions:**

*XPA* expression is an additional independent prognostic biomarker for stratifying GCT patients, allowing for improvements in decision-making on treatment for those at high risk of refractoriness or relapse. In addition, it could represent a novel therapeutic target in GCTs.

## Background

Germ cell tumours (GCTs) represent the most chemosensitive solid malignancy; up to 70–80% of patients with metastatic disease can be cured with the first-line standard-dose cisplatin (CDDP)-based chemotherapy. Nevertheless, 20–30% of patients relapse or do not respond to therapy [1, 2]. About 20–25% of relapsed patients may be cured with salvage conventional or high-dose chemotherapy [1, 3–5]. However, patients who fail to be cured with salvage chemotherapy have a poor prognosis [6, 7].

The main pharmacological target of CDDP is DNA, and therefore DNA alterations represent the key factor determining toxicity. CDDP induces a variety of DNA damage lesions, including DNA interstrand cross-links (ICLs) [8]. ICLs activate the DNA damage response (DDR) and ICL repair pathways, the latter being a highly complex process that involves coordinated action of several DNA repair mechanisms [9], including nucleotide excision repair (NER) pathway. NER employs some 30 DNA repair proteins [10] and mechanistically contributes to ICL repair by mediating lesion recognition and incision. Central to DNA lesion recognition is the xeroderma pigmentosum complementation group A (XPA) protein, which also functions in the DNA lesion verification step and assembly of the NER incision complexes [11]. Incision is mediated by the two structure-specific nucleases, xeroderma pigmentosum complementation group F and G (XPF and XPG, respectively), the former forming a complex with the excision repair cross-complementation group 1 (ERCC1) protein. ERCC1 per se is catalytically inactive and serves to target XPF to different substrates, thereby regulating its availability and activity [12].

Correlation between the chemosensitivity and expression level of the NER factors has attracted research attention. Up-regulation of the NER proteins in tumours has repeatedly been associated with a worse prognosis with decreased ERCC1 mRNA/protein levels leading to an improved response to CDDP-based chemotherapy, and survival, in patients with metastatic lung cancer [13], head and neck squamous cell carcinoma [14], as well as of ovarian [15], gastric [16] and bladder cancers [17]. In GCTs, however, data are far from consistent. While *ERCC1* expression has been shown to correlate with CDDP sensitivity in GCTs [18], no association with patient survival [18] or clinical-pathological features (tumour size and clinical stage) [19] has been reported. Furthermore, *XPA* expression was shown to be highly heterogeneous, with no significant difference detected between CDDP-sensitive tumours and refractory disease. Interestingly, teratomas (TE), and refractory tumours resected in relapse after chemotherapy, have been shown to be strongly XPA positive [20]. In GCT-derived cell lines, no correlation between *XPA* expression and sensitivity to CDDP has been observed and it was therefore concluded that, for patients with newly diagnosed GCTs, XPA detection has no prognostic or predictive value, as it does not play a critical role in overall resistance to treatment [20]. Notably, in vitro cell culture data showed that XPA, ERCC1 and XPF levels are generally lower in GCT cell lines than in cell lines from other tumour types [21, 22].

In this study, we have examined the expression levels of the ERCC1, XPF and XPA proteins in GCT patients and correlated these with clinical-pathological characteristics and therapy outcomes to examine whether increased expression might be associated with inferior survival. We show that the ERCC1, XPF and XPA protein levels are significantly higher in GCTs compared to normal testicular tissues and we report an inverse correlation between *XPA* expression and prognosis in GCT patients. We demonstrate that an increase of the combined expression of the NER proteins (ERCC1, XPF and XPA) also associates with worse overall survival (OS). We propose that increased *XPA* expression, and to a lesser extent of the combined NER, in primary GCTs might facilitate treatment resistance as a consequence of increased DNA repair capacity. Hence, we suggest that NER, particularly XPA, could represent a novel promising therapeutic target in GCTs.

## Methods

### Patients and cell lines

The present study (Protocol IZLO1, Chair: M. Mego) included 207 GCT patients treated between 1999 and 2013 in the National Cancer Institute and/or St. Elisabeth Cancer Institute, Bratislava, Slovakia, with available paraffin-embedded tumour tissue specimen and sufficient follow-up clinical data. Patients with concurrent malignancy other than non-melanoma skin cancer in the previous 5 years were excluded.

NTERA-2 cl.D1 [NT2/D1] cell line is commercially available (ATCC® CRL-1973™). The remaining GCT cell lines, H12.1, H12.1D, H12.1ODM, 2102EP, 1411HP and 1777NRpmet, were kindly provided by Dr. Thomas Mueller, Martin Luther University Halle-Wittenberg, Halle (Saale), Germany [23–27]. CDDP-sensitive (H12.1 and 2102EP) and -resistant (H12.1ODM, 1411HP and 1777NRpmet) GCT cell lines were grown in RPMI-1640 medium supplemented with 5% fetal bovine serum (FBS), penicillin (100 units/ml) and streptomycin (10 μl/ml). NTERA-2 cl.D1 (NT2/D1) GCT cell line was grown in Dulbecco’s modified eagle’s medium supplemented with F-10 nutrient mixture (1:1), 5% FBS, penicillin (100 units/ml) and streptomycin (10 μl/ml). Cell lines were cultivated at 37 °C in 5% CO_2_ atmosphere.

### CDDP treatment

H12.1, H12.1ODM, 1411HP and 1777NRpmet GCT cell lines were cultivated as described above. When the cell cultures reached approximately 80% confluency, cultivation medium was replaced with fresh medium containing 17 μM CDDP. After 2 h treatment, the cells were washed three times with phosphate-buffered saline (PBS) and collected by scrapping.

### Tumour pathology

Pathology review was conducted by two pathologists from the Department of Pathology, Faculty of Medicine, Comenius University, Bratislava, Slovakia.

### Diagnosis and tissue samples

Where available, both tumour and normal testicular tissues were evaluated. The study included tumour specimens from 207 GCT patients before administration of systemic therapy; 200 cases of primary gonadal and 7 cases of extragonadal tumours (5 retroperitoneal and 2 mediastinal). GCTs were classified according to World Health Organization criteria [28]. Normal testicular tissues from non-cancer patients were not available, and therefore normal tissues adjacent to tumours were used (49 samples), as previously described [29, 30].

### Tissue microarray construction

One or two representative tumour areas from each histological subtype of GCTs were identified on H&E sections. Samples from normal testicular tissue were also marked, where available. Sections were matched to their corresponding paraffin blocks (donor blocks), and 3-mm diameter tissue cores were removed from these donor blocks with the multipurpose sampling tool Harris Uni-Core and inserted into the recipient master block. Recipient blocks were cut into 5-μm sections, which were transferred onto coated slides.

### Immunohistochemical staining

Slides were deparaffinized and rehydrated in 10 mM PBS (pH 7.2). The tissue epitopes were unmasked using the Dako PT Link automated water bath heating process. The slides were incubated in Tris-EDTA retrieval solution (10 mM Tris, 1 mM EDTA pH 9.0) (ERCC1 and XPF staining) or citrate retrieval buffer of pH 6.0 (XPA staining) at 98 °C for 20 min. The slides were subsequently incubated for 1 h at room temperature (RT) with the primary mouse monoclonal antibody against ERCC1 (Dako, clone 4F9), mouse monoclonal antibody against XPF (Abcam [SPM228]: ab17798) or mouse monoclonal antibody against XPA (Abcam [12F5]: ab2352) diluted 1:100 in Dako REAL antibody diluent (Dako) and immunostained using anti-mouse/anti-rabbit immuno-peroxidase polymer (EnVision FLEX/HRP, Dako) at RT for 30 min, according to the manufacturer’s instructions. For visualization, the slides were treated with diaminobenzidine substrate-chromogen solution (DAB, Dako) for 5 min. Finally, the slides were counterstained with haematoxylin. Appendix or tonsil tissue samples were used as positive controls for ERCC1 or XPA and XPF, respectively. The same tissues with omitted primary antibodies served as a negative control.

### Immunohistochemical stain scoring

Tumour cores were independently assessed by two pathologists who were blinded to the clinical-pathological data. In cases of disagreement, a consensus was found. The expression levels of the NER proteins were scored by a weighted histoscore (HS), which accounts for both the extent of cell staining and the staining intensity [31]. The percentage of positively stained cells was calculated and the average intensity of positively stained cells was given a score from 0 to 3 (0 = no staining, 1 = weak, 2 = intermediate and 3 = strong staining). The HS was then calculated by multiplying the percentage score by the intensity score, to yield a minimum value of 0 and a maximum value of 300. If multiple histological subtypes were present in a sample, the greater HS was taken. Based on the HS, the NER protein expression levels were graded as “low” (≤ 150) or “high” (> 150). The combined NER expression level was calculated as sum of weighted HS for each individual NER protein to yield a minimum value of 0 and a maximum value of 900. In a similar manner to the expression level of each individual NER protein, the combined NER expression level was graded as “low” (≤ 450) or “high” (> 450).

### mRNA expression evaluation

Total RNA was extracted using TRI Reagent solution (Life Technologies) and quantified using MaestroNano Spectrophotometer (Applied Biological Materials Inc.) and Qubit fluorometer (Qubit® RNA HS Assay Kit, Life Technologies). Differential expression of the *XPA*, *ERCC1* and *XPF* genes was evaluated by qRT-PCR. Briefly, cDNA was synthesized, using First-Strand cDNA Synthesis System from Central European Biosystems, from 1.5 μg of total RNA in 20 μl reactions containing 2 μl of 10x MuLV buffer, 1 μM of p(dN)6 primer, 0.1 mM of dNTP mix and 100 units of MuLV reverse transcriptase. These were incubated at 42 °C for 1 h followed by enzyme inactivation at 95 °C for 5 min. Real-time PCR detection and quantification of *XPA*, *XPF* and *ERCC1* expression was performed using SYBR Premix Ex Taq II (Tli RNaseH Plus), ROX plus (Takara) and primers listed in Additional file 1: Table S1. Ct values were normalized against the *PGK1* reference gene, which is stably expressed across all GCT cell lines tested.

### Protein expression evaluation

Protein expression analyses were performed using cell lysates prepared in ice-cold RIPA buffer (50 mM Tris-HCl, pH 7.5; 150 mM NaCl; 5 mM EDTA; 1% Triton X-100; 0.5% NaDOC; 0.1% SDS) containing MS-SAFE Protease and Phosphatase Inhibitor (Sigma Aldrich). Primary antibodies against XPA, phosphorylated XPA at S196 [pXPA(S196)] and XPF were purchased from Thermo Fisher Scientific. The ERCC1 antibody was obtained from Santa Cruz Biotechnology. The clarified protein lysates were diluted in 5x SDS-PAGE sample loading buffer (250 mM Tris·HCl, pH 6.8, 10% SDS, 30% (v/v) glycerol, 10 mM DTT, 0.05% (w/v) bromophenol blue), boiled at 95 °C for 10 min, and resolved on Mini-PROTEAN® TGX Stain-Free™ Precast Gels (BioRad) at 120 V. To quantify total protein, gels were activated by exposure to ultraviolet light (UV; 302 nm) for 5 min and visualized using an Amersham Imager 600 (GE Healthcare). The resolved proteins were transferred onto Amersham Protean 0.1 μm nitrocellulose membrane (Sigma Aldrich) in transfer buffer (25 mM Tris base, 190 mM glycine, 20% methanol) at 70 V in a wet blotting apparatus for 1 h at 4 °C. The membrane was blocked with 5% skimmed milk powder diluted in Tris-buffered saline (TBS; 10 mM Tris base, pH 7.4; 150 mM NaCl) at RT for 1 h. Membranes were incubated with primary antibodies diluted in 2.5% skimmed milk in TTBS (TBS containing 0.05% Tween 20) [anti-XPA 1: 1,000; anti-pXPA(S196) 1: 500; anti-XPF 1: 1,000 and anti-ERCC1 1:750] at RT for 1 h with shaking and washed twice in TTBS for 5 min. The membrane was then incubated with goat anti-mouse HRP-conjugated secondary antibody (1: 20,000) (Santa Cruz Biotechnology) at RT for 1 h with shaking. The protein bands were visualized using SuperSignal™ West Femto Maximum Sensitivity chemiluminescence substrate (Thermo Fisher Scientific) and the Odyssey® Fc Imaging System (Li-COR). Protein quantification was performed using the Li-COR Image Studio software normalized to the total protein content.

Quantification of pXPA(S196) expression was performed by dot blot analysis. In a typical dot blot experiment, 20 μg of protein extract were mixed with an equal volume of nanopure H_2_O and blotted onto pre-wetted Immobilon-FL membrane (Sigma Aldrich) (0.8 μl/dot) and allowed to dry. After re-wetting in methanol, the membrane was stained with REVERT total protein stain (Li-COR) and then imaged with the Odyssey® Fc Imaging System in the 700 nm channel. The membrane was then washed with TBS and blocked for 1 h in TTBS containing 2% albumin. The blot was incubated at 4 °C overnight with the pXPA(Ser196) primary antibody diluted 1:500, followed by 2 h in secondary antibody, goat anti-rabbit HRP-conjugated antibody (1:20,000), and chemiluminescence detection (SuperSignal™ West Femto Maximum Sensitivity Substrate). Dot blots were analyzed using Li-COR Image Studio software. For quantification, intensity of each dot obtained by chemiluminescence was normalized to the 700 nm intensity of the corresponding sample, representing the total protein content.

### Statistical analysis

Since the distribution of weighted HS for expression of the NER proteins was significantly different from the normal distribution (the Shapiro-Wilk test), we used non-parametric tests for analyses. Analyses of differences in distributions of the expression levels of the NER proteins between the two groups of patients were performed using the Mann-Whitney U test, while multiple groups were compared by the Kruskal-Wallis test.

Median follow-up period was calculated as a median observation time among all patients and among those still alive at the time of their last follow-up. OS was calculated from the date of orchiectomy or tumour biopsy to the date of death or last follow-up. OS was estimated using the Kaplan-Meier product limit method and compared by the log-rank test. All reported *p* values were two-sided. Statistical analyses were performed using NCSS 11 statistical analysis software (J. Hintze, Kaysville, Utah, USA).

For statistical analysis of the mRNA and protein expression data, SigmaPlot 12.5 was used. Normality of distribution was tested by the Shapiro-Wilk and Kolmogorov-Smirnov tests. Relative quantification of the mRNA expression was calculated with 2^-ΔΔCt^ method, which represents relative fold changes of the mRNA expression. Therefore, ΔΔCt = ΔCt (CDDP-resistant cell line) - ΔCt (CDDP-sensitive cell line). Analysis of the significance of fold change in the mRNA expression between the studied groups was applied to the ΔCt values. If normally distributed, mRNA and protein expression data were tested by the Student’s t-test or analysis of variance (ANOVA) with the Bonferroni’s or Tukey’s test for multiple comparisons. The non-parametric Mann-Whitney Rank Sum test and the Kruskal-Wallis One Way ANOVA with the Tukey’s test for multiple comparisons were used for non-normally distributed data. All tests were two-tailed, performed at the significance level α = 0.05.

For all analyses, *p* <  0.05 was considered statistically significant (*p < 0.05; **p < 0.01, ***p < 0.005).

## Results

### The expression of the NER proteins in GCT patients

Patients’ characteristics are summarized in Additional file 2: Table S2. The majority of patients had non-seminomatous primary testicular tumour and a good prognosis according to the IGCCCG risk group. All patients were treated with CDDP-based chemotherapy. Tumour specimens included 38 pure seminomas and 169 non-seminomas.

We observed significantly higher expression of all three evaluated NER proteins in GCTs in comparison to normal testicular tissues (Table 1). The highest *XPA* and *ERCC1* expression was found in TE, with decreasing amounts in yolk sac tumours and choriocarcinoma. In contrast, lowest expression was detected in seminoma and embryonal carcinoma. Interestingly, while *XPF* expression was considerably higher than *ERCC1* or *XPA* expression (in both tumourous and normal tissue) there was no significant difference in *XPF* expression across all GCT histological subtypes (Table 1).

**Table 1:** The expression of the NER proteins in different histological subtypes of primary GCTs (*n* = 207)

		ERCC1				XPF				XPA			
Histological subtype	*N*	Mean HS	SEM	Median	*p* value^a^	Mean HS	SEM	Median	*p* value	Mean HS	SEM	Median	*p* value
Normal testis	49	0.0	0.0	0	NA	29.4	7.0	0	NA	0.0	0.0	0	NA
Germ cell tumours^b^	207	13.5	2.9	0.0	< **0.01**	213.4	6.2	220.0	< **0.01**	51.6	4.5	20.0	< **0.01**
Seminoma	64	2.2	1.6	0	< **0.01**	182.1	12.4	200	< **0.01**	41.4	7.3	10	< **0.01**
Embryonal carcinoma	118	2.2	0.9	0	< **0.01**	224.0	6.9	220	< **0.01**	19.9	3.4	0	< **0.01**
Yolk sac tumour	30	14.0	6.7	0	< **0.01**	202.4	20.4	210	< **0.01**	87.4	15.0	50	< **0.01**
Choriocarcinoma	15	15.3	7.1	0	< **0.01**	181.7	24.1	200	< **0.01**	53.8	16.2	50	< **0.01**
Teratoma	41	56.0	11.6	20	< **0.01**	186.9	13.2	180	< **0.01**	103.4	10.6	90	< **0.01**
GCNIS	71	4.3	4.2	0	< **0.01**	193.1	13.0	200	< **0.01**	7.6	3.3	0	< **0.01**

Boldface *p* value denotes statistical significance < 0.05

*NA* Not applicable; *GCNIS* Germ cell neoplasia in situ; *SEM* Standard error of the mean; *HS* Histoscore

^a^Compared to normal testicular tissue

^b^The highest NER expression in any histologic subtype in case of mixed GCTs

Correlation between expression of individual NER proteins and clinical-pathological characteristics is shown in Table 2. The expression levels of all three NER proteins were higher in non-seminomas compared to seminomas, although for *ERCC1* the difference was not statistically significant. Higher *XPA* expression was observed in patients with more advanced disease including IGCCCG poor prognosis group, lung, liver and other non-pulmonary visceral metastases, as well as with increasing S stage. Primary extragonadal GCTs had higher XPA protein levels compared to primary TGCTs; however, this difference was not statistically significant. Higher *ERCC1* expression correlated with IGCCCG risk group and S stage. *XPF* expression was not associated with any observed parameter (Table 2).

**Table 2:** Patient’s characteristics according to the expression of individual NER proteins in primary tumour (*n* = 207)

		ERCC1				XPF				XPA			
Variable	*N*	Mean HS	SEM	Median	*p* value	Mean HS	SEM	Median	*p* value	Mean HS	SEM	Median	*p* value
All patients	207	13.5	2.9	0.0	NA	213.4	6.2	220.0	NA	51.6	4.5	20.0	NA
Histology													
Seminoma	38	1.9	6.8	0.0	0.0807	163.0	13.8	200.0	^***^0.0015	31.9	10.5	5.0	^*^0.0196
Non-seminomaNNNN Non-seminona	169	15.9	3.2	0.0		224.6	6.6	240.0		56.0	5.0	30.0	
Tumour primary													
Primary TGCTs	200	13.5	2.9	0.0	0.4771	212.2	6.2	210.0	0.2250	50.8	4.6	20.0	0.0676
Extragonadal GCTs	7	12.9	15.6	0.0		248.3	35.6	300.0		79.2	26.3	80.0	
IGCCCG risk group													
Good prognosis	158	9.9	3.3	0.0	^*^0.0322	210.5	7.0	215.0	0.3983	44.7	5.0	10.0	^***^0.0002
Intermediate	23	21.3	8.5	0.0		239.8	18.2	265.0		60.2	13.1	50.0	
Poor prognosis	26	28.2	8.2	0.0		206.0	17.4	200.0		91.8	13.4	75.0	
Number of metastatic sites													
0	54	13.4	5.6	0.0	0.5566	216.3	11.9	210.0	0.8651	49.3	8.7	20.0	0.0628
1 to 2	124	11.9	3.7	0.0		210.3	8.0	210.0		47.0	5.7	20.0	
> 3	29	20.6	7.9	0.0		220.2	16.6	250.0		82.5	13.0	55.0	
Retroperitoneal lymph nodes metastases													
Absent	61	13.4	5.3	0,0	0.5226	215.4	11.2	210.0	0.8157	53.9	8.3	20.0	0.7993
Present	146	13.5	3.4	0.0		212.4	7.4	220.0		50.6	5.4	20.0	
Mediastinal lymph nodes metastases													
Absent	189	12.8	3.0	0.0	0.9408	213.3	6.5	215.0	0.9501	50.2	4.7	20.0	0.4597
Present	18	21.2	10.0	0.0		213.1	20.6	225.0		72.7	16.6	75.0	
Lung metastases													
Absent	160	10.9	3.3	0.0	0.1664	208.6	7.0	205.0	0.1478	47.6	5.1	10.0	^*^0.0252
Present	47	22.3	6.0	0.0		229.2	12.8	257.5		67.0	9.7	50.0	
Liver													
Absent	197	12.7	2.9	0.0	0.0510	213.0	6.3	210.0	0.8140	49.1	4.6	20.0	^***^0.0037
Present	10	28.0	13.0	0.0		219.0	27.7	250.0		110.6	21.1	120.0	
Non-pulmonary visceral metastases													
Absent	194	13.2	3.0	0.0	0.2293	213.1	6.4	210.0	0.9441	49.0	4.6	20.0	^***^0.0032
Present	13	16.5	11.4	0.0		216.2	24.3	230.0		101.4	19.1	70.0	
S stage													
0	78	9.3	4.6	0.0	^*^0.0455	198.9	9.7	200.0	0.3526	38.5	7.1	5.0	^***^0.0004
1	78	6.9	4.6	0.0		218.9	9.8	240.0		51.6	7.1	20.0	
2	29	29.7	7.5	0.0		232.9	16.0	250.0		61.9	12.1	50.0	
3	16	36.6	10.1	0.0		226.0	22.2	240.0		102.3	16.2	100.0	
unknown	6												

Boldface *p* value denotes statistical significance < 0.05: ^*^p < 0.05; ^**^p < 0.01, ^***^p < 0.005

*NA* Not applicable; *TGCT* Testicular germ cell tumour; *GCT* Germ cell tumour; *IGCCCG* International Germ Cell Consensus Classification Group; *HS* Histoscore; *SEM* Standard error of the mean

**Fig. 1:**
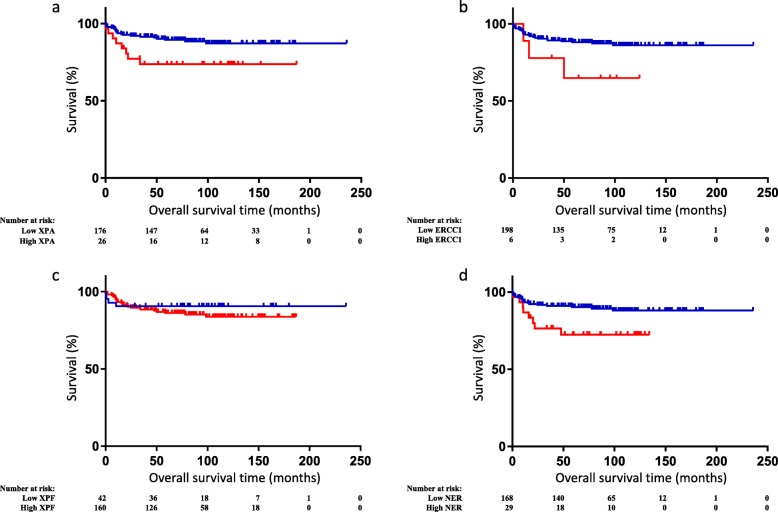
Kaplan-Meier analysis illustrating OS based on (**a**) *XPA*, (**b**) *ERCC1*, (**c**) *XPF* and (**d**) combined NER (*ERCC1*, *XPF* and *XPA*) expression in GCT patients (*n* = 207). (**a**) HR = 0.38, 95% CI: 0.12–1.23, *p* = 0.0228; (**b**) HR = 0.35, 95% CI: 0.03–3.56, *p* = 0.1295; (**c**) HR = 0.65, 95% CI: 0.26–1.64, *p* = 0.4250; and (**d**) HR = 0.36, 95% CI: 0.12–1.09, *p* = 0.0109. Blue and red lines represent low and high expression, respectively

The median follow-up time was 81.8 months (0.4–235.8) for all patients and 92.9 months (7.1–235.8) for patients who survived. During follow-up, 28 patients died. All observed deaths were due to testicular cancer. Estimated 5-year OS was 87.1%. Patients with low *XPA* expression had significantly better OS than patients with high *XPA* expression (hazard ratio [HR] = 0.38, 95% confidence interval [CI]: 0.12–1.23, *p* = 0.0228) (Fig. 1a). The differences were more pronounced in the non-seminomatous group and in patients with metastatic disease (HR = 0.36, 95% CI: 0.11–1.19, *p* = 0.0189 and HR = 0.34, 95% CI: 0.1–1.15, *p* = 0.0102, respectively). Similarly, OS was inversely associated with *ERCC1* (HR = 0.35, 95% CI: 0.03–3.56 *p* = 0.1295) and *XPF* (HR = 0.65, 95% CI: 0.26–1.64, *p* = 0.4250) expression (Fig. 1b and c, respectively), though these data were not statistically significant. An increase in combined expression of all three NER proteins (ERCC1 + XPF + XPA) was observed in non-seminomatous histological subtype, primary extragonadal tumour, IGCCCG poor prognosis group, > 3 metastatic sites, lung metastasis and increased S stage, and was associated with worse OS (HR = 0.36, 95% CI: 0.12–1.09, *p* = 0.0109) (Table 3, Fig. 1d).

**Table 3:** Patient’s characteristics according to the expression of combined NER (ERCC1, XPF and XPA) in primary tumour (*n* = 207)

		Combined NER			
Variable	*N*	Mean HS	SEM	Median	*p* value
All patients	207	282.6	10.1	280.0	NA
Histology					
Seminoma	38	202.2	22.9	205.0	^***^0.0004
Non-seminomaNNNN Non-seminona	169	300.6	10.8	300.0	
Tumour primary					
Primary TGCTs	200	279.9	10.2	270.0	^*^0.0500
Extragonadal GCTs	7	387.0	63.4	360.0	
IGCCCG risk group					
Good prognosis	158	266.4	11.3	260.0	^**^0.0062
Intermediate	23	321.3	29.1	320.0	
Poor prognosis	26	358.3	30.4	345.0	
Number of metastatic sites					
0	54	279.0	19.2	250.0	^*^0.0471
1 to 2	124	271.0	12.9	270.0	
> 3	29	348.3	28.8	330.0	
Retroperitoneal lymph nodes metastases					
Absent	61	282.7	18.3	250.0	0.6121
Present	146	282.5	12.2	287.5	
Mediastinal lymph nodes metastases					
Absent	189	277.9	10.5	270.0	0.1142
Present	18	339.7	36.6	310.0	
Lung metastases					
Absent	160	268.8	11.3	252.5	^***^0.0048
Present	47	331.9	21.4	325.0	
Liver					
Absent	197	278.5	10.3	270.0	0.0584
Present	10	368.3	47.1	350.0	
Non-pulmonary visceral metastases					
Absent	194	278.3	10.4	270.0	0.0661
Present	13	354.5	42.7	345.0	
S stage					
0	78	246.7	15.5	232.5	^***^0.0004
1	78	283.1	15.8	285.0	
2	29	335.0	26.3	345.0	
3	16	385.7	36.5	362.5	
unknown	6				

Boldface *p* value denotes statistical significance < 0.05: ^*^p < 0.05; ^**^p < 0.01, ^***^p < 0.005

*NA* Not applicable; *TGCT* Testicular germ cell tumour; *GCT* Germ cell tumour; *IGCCCG* International Germ Cell

Consensus Classification Group; *HS* Histoscore; *SEM* Standard error of the mean

Multivariate analysis revealed that XPA protein level is significantly associated with OS independent of IGCCCG risk group (Table 4), indicating that XPA is promising IGCCCG-independent prognostic factor for OS in GCTs.

**Table 4:** Prognostic value of *XPA* expression

Variable	OS	
	Univariate analysis HR (95% CI) *p* value	Multivariate analysis HR (95% CI) *p* value
*XPA* expression		
Low vs high	0.38 (0.12–1.23) *p* = **0.023**	0.42 (0.18–1.01) *p* = 0.053
IGCCCG risk group		
Good vs intermediate/poor	0.12 (0.05–0.30) *p* < **0.00001**	0.11 (0.05–0.26) *p* = **0.0001**

*OS* overall survival, *HR* hazard ratio, *CI* confidence interval, *IGCCCG* International Germ Cell

Consensus Classification Group

Boldface *p* value denotes statistical significance < 0.05

### The expression of the NER factors in GCT cell lines

**Fig. 2:**
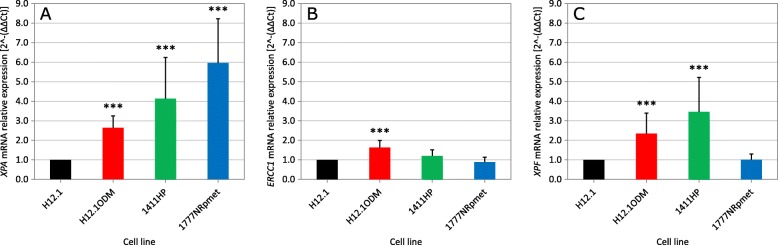
Comparison of the (**a**) *XPA*, (**b**) *ERCC1* and (**c**) *XPF* mRNA expression between CDDP-resistant (H12.1ODM, 1411HP and 1777NRpmet) and -sensitive (H12.1) GCT cell lines. Error bars represent the standard deviation of three technical replicates of three biological replicates. * *p* ≤ 0.01, ** *p* ≤ 0.005, *** *p* ≤ 0.001

We wanted to further study the association between NER expression and prognosis for GCT patients in vitro by examining mRNA and protein levels of ERCC1, XPF and XPA in well-characterized CDDP-resistant and -sensitive GCT cell lines [23, 24]. As shown in Fig. 2a and Additional file 3: Figure S1A, *XPA* mRNA expression was significantly higher in all CDDP-resistant compared to -sensitive GCT cell lines, while *ERCC1* mRNA expression levels were either unchanged or slightly higher in CDDP-resistant vs -sensitive lines (Fig. 2b and Additional file 3: Figure S1B). The picture is less clear for *XPF* expression; while CDDP-resistant cell lines H12.1ODM and 1411HP showed a statistically significant increase in *XPF* expression compared with CDDP-sensitive cell lines, no difference was seen for the cell line 1777NRpmet (Fig. 2c and Additional file 3: Figure S1C).

**Fig. 3:**
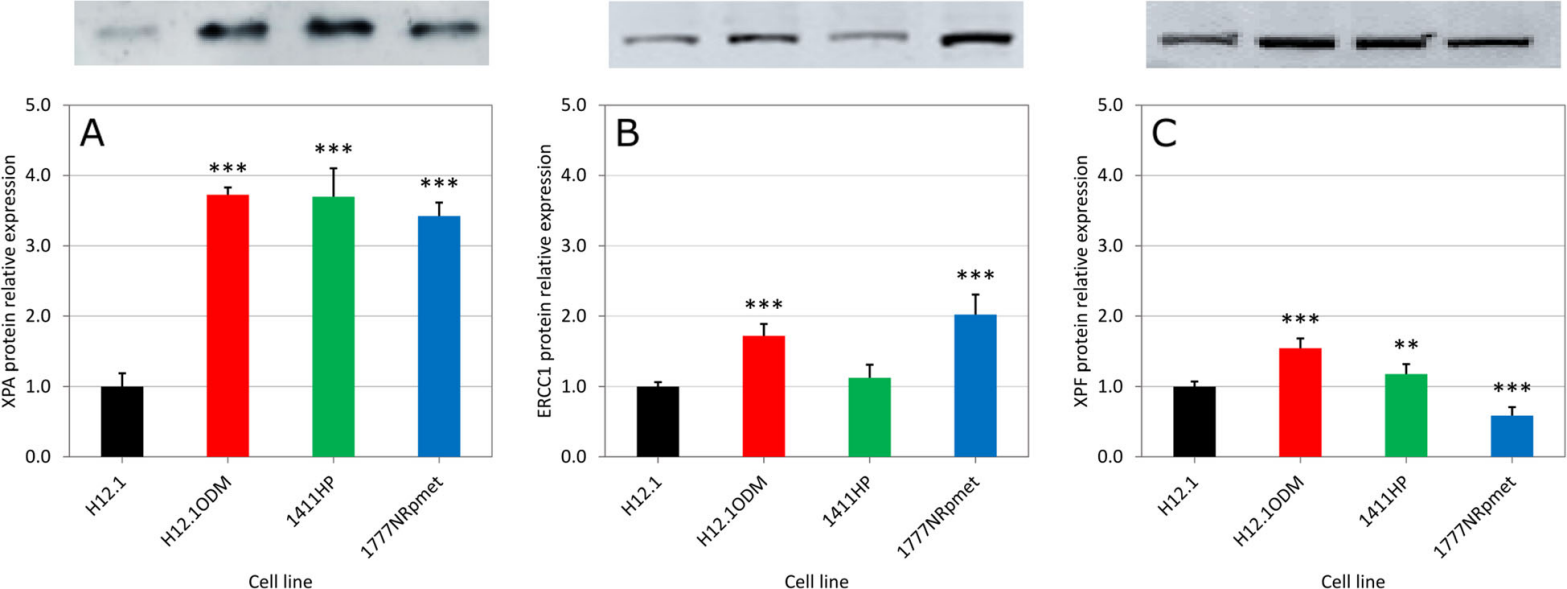
Comparison of the (**a**) XPA, (**b**) ERCC1 and (**c**) XPF protein expression between CDDP-resistant (H12.1ODM, 1411HP and 1777NRpmet) and -sensitive (H12.1) GCT cell lines. Upper panels show representative Western blots. Lower panels (graphs) are quantitative analyses of three technical replicates of three biological replicates with error bars representing the standard deviation. * *p* ≤ 0.01, ** *p* ≤ 0.005, *** *p* ≤ 0.001

Relative ERCC1, XPF and XPA protein expression levels in CDDP-resistant compared to -sensitive GCT cell lines are summarized in Fig. 3 and Additional file 4: Figure S2. With the exception of 1411HP vs NTERA-2 pairwise combination, the XPA protein levels were remarkably higher in CDDP-resistant compared to -sensitive GCT cell lines (Fig. 3a and Additional file 4: Figure S2A). Similarly, ERCC1 protein levels were elevated in CDDP-resistant compared with -sensitive GCT cell lines, however, this was less pronounced (Fig. 3b and Additional file 4: Figure S2B). However, no direct correlation can be drawn from XPF protein levels and CDDP-resistance (Fig. 3c and Additional file 4: Figure S2C). Together, these data suggest that overexpression of NER factors, particularly *XPA*, correlates with response to CDDP, mirroring our clinical data. Based on our findings, we propose that one of the fundamental mechanisms of CDDP resistance in GCTs is up-regulation of XPA and, by inference, the whole NER pathway.

### The role of XPA phosphorylation in establishing CDDP resistance

XPA has previously been shown to interact with, and be phosphorylated by, ataxia telangiectasia-mutated and Rad3-related (ATR) checkpoint kinase at S196 [32, 33], however the role of this event is still rather elusive. It is possible that this phosphorylation event, which requires Sirtuin 1-dependent deacetylation of XPA, leads to cytosolic-to-nuclear translocation and stabilization (by inhibiting ubiquitination and subsequent degradation) of XPA [34–37]. To further understand the role of XPA in the CDDP resistance mechanism in GCTs, we examined the possibility that S196 phosphorylation plays a role in this process.

**Fig. 4:**
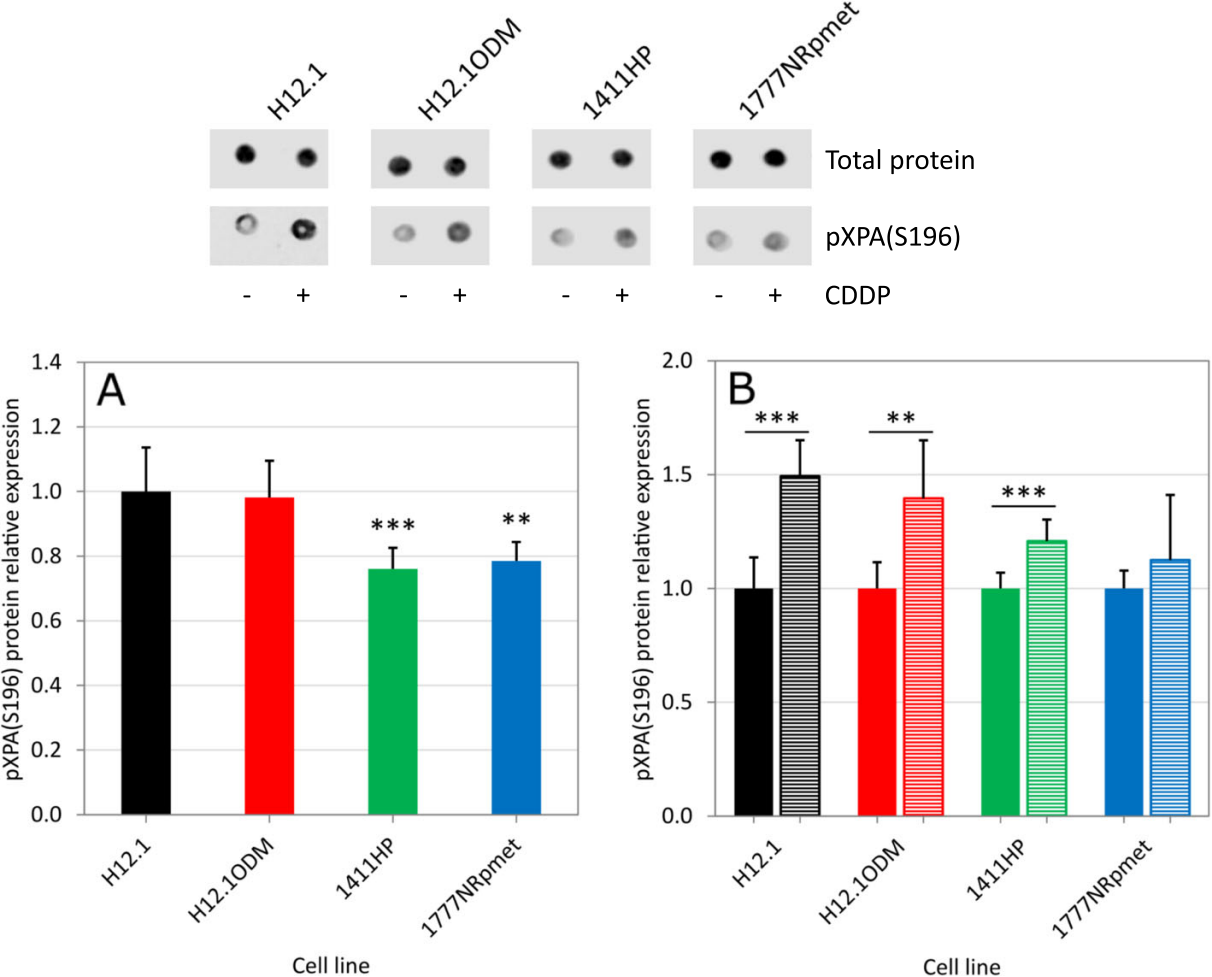
Expression of (**a**) phosphorylated XPA and (**b**) its increase after CDDP treatment in CDDP-resistant (H12.1ODM, 1411HP and 1777NRpmet) and -sensitive (H12.1) GCT cell lines. CDDP-treated samples are shown in hatched columns. Upper panel shows representative dot blot. Lower panels (graphs) are quantitative analyses of three technical replicates of three biological replicates with error bars representing the standard deviation. * *p* ≤ 0.01, ** *p* ≤ 0.005, *** *p* ≤ 0.001

While we observed a 3- to 4-fold increase in XPA protein in CDDP-resistant compared to -sensitive GCT cell lines (Fig. 3a), no difference was seen in XPA phosphorylation (Fig. 4a). CDDP treatment led to an increase in XPA phosphorylation, in a similar manner to UV exposure [32], however there was little difference in this response between CDDP-resistant and -sensitive cell lines tested (Fig. 4b). These data indicate that (i) XPA phosphorylation does not have an impact on increase of the total XPA protein level, (ii) CDDP-induced DNA damage may represent a relatively weak signal leading to ATR-mediated XPA phosphorylation, and (iii) XPA phosphorylation does not play a role in the CDDP resistance mechanism in GCTs.

## Discussion

Resistance to CDDP poses a serious problem in patient management as it leads to poor prognosis in many malignancies. GCTs are among the most CDDP-sensitive malignity, and therefore represent a highly valuable model for the study of CDDP resistance mechanism.

CDDP induces a wide variety of DNA lesions, and therefore cellular response to this drug is multifaceted. Central to the repair of such lesions is the Fanconi anemia pathway, a highly complex DDR and repair pathway that coordinates the action of a plethora of DNA repair mechanisms, including NER, required to remove CDDP-induced DNA damage. NER requires many DNA repair enzymes that act sequentially to remove bulky DNA adducts, such as ICLs. NER contributes significantly to the cellular response to ICL-inducing agents, such as CDDP, widely used in anti-cancer chemotherapy regimens, thereby highlighting the clinical importance of this repair pathway for therapeutic outcome.

It has been hypothesized that CDDP sensitivity in GCTs may be caused by decreased capacity of NER, as a consequence of a reduced expression of its key components. Indeed, the expression of *XPA* and components of the ERCC1-XPF nuclease have been found to be lower in cell lines derived from GCTs compared to those from other tumour types [21, 22]. This difference, however, does not appear to affect the cells ability to perform NER [38].

The purpose of the present study was to determine the expression levels of the key NER factors, ERCC1, XPF and XPA, in a substantial group of GCTs displaying a variety of histological subtypes. We show significantly increased expression of these factors in all histological subtypes of GCTs compared to normal testicular tissues, which is consistent with previously reported data [19]. Notably, while *XPF* expression was consistent across all GCT histological subtypes, expression of *ERCC1* and *XPA* varied greatly, with increased expression in the most differentiated TE, suggesting that the process of differentiation of germ cells to somatic structures may require NER factors. These mature TE are among the most CDDP-resistant histological subtypes.

We demonstrate the prognostic value of *XPA* expression on OS. In addition, we disclose a statistically significant correlation between the increased *XPA* expression and poor prognostic features in GCTs, including non-seminomatous histology, the IGCCCG poor prognosis group, presence of lung, liver and/or other non-pulmonary visceral metastases and high serum tumour markers. No other NER factor examined showed such a strong correlation with clinical-pathological characteristics in GCTs, although the whole NER pathway disclosed better associations compared to XPA alone. The question now remains whether single NER factors or a combination of many NER factors would provide more precise clinical information, as these two options may not lead to the same treatment outcomes.

XPA has numerous binding partners (reviewed in [11]) and is known to exhibit NER-independent cellular functions, including a role in bi-directional transcriptional change in a subset of genes [39]. The phenotype of XPA-deficient cells would also suggest a critical role for XPA outside NER [40]. Of these functions, regulation of steroid hormone metabolism may be of great importance in GCTs. Having a regulatory role in metabolism of testosterone, XPA may contribute to pathological imbalance of the hormone during early (fetal) development, resulting in GCTs in later developmental stages. Indeed, higher testosterone levels detected at birth were found to be associated with increased GCT risk among adolescents (15–19 years) [41]. XPA is involved in nuclear-mitochondrial crosstalk that is critical for the maintenance of mitochondrial homeostasis [40]. Biochemically functional mitochondria are critical for the synthesis of pregnenolone, a precursor for steroid hormones. XPA-mediated control of mitochondrial health may, therefore, be a mechanism contributing to the development and progression of GCTs. Studies into any association between XPA and testosterone levels would be worthwhile.

ERCC1 has previously been shown to influence CDDP sensitivity in GCTs [18]. Here, we confirm that increased expression of *ERCC1* correlates with poor prognosis and an increased S stage of disease. However, we did not find any statistically significant correlation between *ERCC1* expression and OS, in agreement with previously published data [18]. Therefore, ERCC1 on its own does not seem to be a reliable prognostic marker in GCTs.

The CDDP response in GCT patients and cell lines are both highly dependent on the expression levels of NER factors and GCT cell lines can, therefore, be used as a reliable model for studying CDDP resistance mechanisms. Herein, we revealed that the levels of ERCC1, XPF and XPA protein correspond with expression and not protein stability. We ruled out a possibility that S196 phosphorylation significantly influences the resulting XPA protein levels. Undoubtedly, further work is required to fully understand the mechanisms driving NER expression in CDDP resistance. Once these mechanisms are sufficiently understood, their therapeutic targeting could be investigated.

## Conclusions

We show significantly higher expression of the three key NER proteins, ERCC1, XPF and XPA, in GCTs compared to normal testicular tissue. Moreover, we report a correlation between higher *XPA* expression in primary tumour tissues and inferior outcome in GCT patients: *XPA* expression is higher in patients with more advanced disease and poor prognostic features, suggesting a possibility that its increased level may ensure CDDP chemoresistance in primary GCTs and subsequent tumour dissemination and disease progression. Importantly, a correlation between *XPA* expression and CDDP-based treatment response obtained using patients’ samples was well-mirrored in GCT cell lines. In contrast to what we expected, S196 phosphorylation does not seem to have an essential role in ensuring high XPA levels in CDDP-resistant GCT cell lines. Instead, accumulation of XPA protein is achieved by up-regulation of expression. In summary, we propose that the expression level of XPA, as well as NER as a whole, represent additional prognostic biomarkers for stratifying GCT patients to optimize the disease management. In addition, they potentially constitute promising therapeutic targets in this malignity.

## Supplementary information

**Additional file 1: Table S1.** Primers used in this study

**Additional file 2: Table S2.** Patients’ characteristics (*n* = 207)

**Additional file 3: Figure S1.** Comparison of the (**a**) *XPA*, (**b**) *ERCC1* and (**c**) *XPF* mRNA expression between CDDP-resistant (1411HP and 1777NRpmet) and -sensitive (2102EP and NTERA-2) GCT cell lines. Error bars represent the standard deviation of three technical replicates of three biological replicates. * *p* ≤ 0.01, ** *p* ≤ 0.005, *** *p* ≤ 0.001

**Additional file 4: Figure S2.** Comparison of the (**a**) XPA, (**b**) ERCC1 and (**c**) XPF protein expression between CDDP-resistant (1411HP and 1777NRpmet) and -sensitive (2102EP and NTERA-2) GCT cell lines. Upper panels show representative Western blots. Lower panels (graphs) are their quantitative analyses with error bars representing the standard deviation of three technical replicates of three biological replicates. * *p* ≤ 0.01, ** *p* ≤ 0.005, *** *p* ≤ 0.001

## Data Availability

All data generated or analysed during this study are included in this published article.

## Abbreviations

AFP: α-fetoprotein
ANOVA: Analysis of variance
ATR: Ataxia telangiectasia-mutated and Rad3-related
CDDP: Cisplatin
CI: Confidence interval
DDR: DNA damage response
ERCC1: Excision repair cross-complementation group 1
FBS: Fetal bovine serum
GCNIS: Germ cell neoplasia in situ
GCT: Germ cell tumour
HR: Hazard ratio
HS: Histoscore
ICL: Interstrand cross-link
IGCCCG: International Germ Cell Cancer Collaborative Group
LDH: Lactate dehydrogenase
NER: Nucleotide excision repair
OS: Overall survival
PBS: Phosphate-buffered saline
PGK1: Phosphoglycerate kinase 1
pXPA(S196): XPA phosphorylated at serine 196
qRT-PCR: Quantitative reverse transcription polymerase chain reaction
RT: Room temperature
SEM: Standard error of the mean
TBS: Tris-buffered saline
TGCT: Testicular germ cell tumour
XPA: Xeroderma pigmentosum complementation group A
XPF: Xeroderma pigmentosum complementation group F
XPG: Xeroderma pigmentosum complementation group G
β-HCG: β-human chorionic gonadotropin
