# Supplementary material for: Increased levels of XPA might be the basis of cisplatin resistance in germ cell tumours
Source: BMC Cancer. 2020 Jan 6;20:17. doi: 10.1186/s12885-019-6496-1 (PMC6945513; doi:10.1186/s12885-019-6496-1)
Supplement: Supplementary file 1 — Additional file 1: Table S1. Primers used in this study [file 12885_2019_6496_MOESM1_ESM.docx]

**Table S1** Primers used in this study

| Primer name (the targeted gene) | Sequence (5' to 3') |
| --- | --- |
| ***XPA_FW*** | TCACAATGGGGTGATATGAAACTC |
| ***XPA_REV*** | CTGTCGGACTTCCTTTGCTTC |
| ***XPF_FW*** | AACCTTTGTTCGGCAGCTTG |
| ***XPF_REV*** | GCGTTGTTCCTCAGTTGAACC |
| ***ERCC1_ FW*** | CTGCTTGTCCAGGTGGATG |
| ***ERCC1 _REV*** | GCTGGTTTCTGCTCATAGGC |
| ***PGK1_FW*** | TGGAGCTCCTGGAAGGTAAAG |
| ***PGK1_REV*** | AAGTTGACTTAGGGGCTGTGC |
