# Supplementary material for: Increased levels of XPA might be the basis of cisplatin resistance in germ cell tumours
Source: BMC Cancer. 2020 Jan 6;20:17. doi: 10.1186/s12885-019-6496-1 (PMC6945513; doi:10.1186/s12885-019-6496-1)
Supplement: Supplementary file 2 — Additional file 2: Table S2. Patients’ characteristics (n = 207) [file 12885_2019_6496_MOESM2_ESM.docx]

**Table S2** Patients’ characteristics (*n* = 207)

| Variables | *N* | % |
| --- | --- | --- |
| All | 207 | 100 |
| Age (years) |  |  |
| Median (range) | 30 | 17-67 |
| Histology |  |  |
| Seminoma | 38 | 18.4 |
| Non-seminoma | 169 | 81.6 |
| Primary tumor |  |  |
| Gonadal | 200 | 96.6 |
| Retroperitoneal | 5 | 2.4 |
| Mediastinal | 2 | 1.0 |
| IGCCCG risk group |  |  |
| Good prognosis | 158 | 76.3 |
| Intermediate prognosis | 23 | 11.1 |
| Poor prognosis | 26 | 12.6 |
| Sites of metastases |  |  |
| Retroperitoneum | 146 | 70.5 |
| Mediastinum | 18 | 8.7 |
| Lungs | 47 | 22.7 |
| Liver | 10 | 4.8 |
| Brain | 1 | 0.5 |
| Visceral non-pulmonary metastases | 13 | 6.3 |
| Number of metastatic sites |  |  |
| 0 | 54 | 26.1 |
| 1 | 96 | 46.4 |
| 2 | 28 | 13.5 |
| >3 | 29 | 14.0 |
| Mean (range) of pretreatments markers |  |  |
| AFP mIU/ml | 7.1 | 0 - 60,570 |
| β-HCG IU/ml | 3.0 | 0 - 929,000 |
| LDH (mkat/l ) | 6.3 | 2 - 89 |
| *β-HCG* β-human chorionic gonadotropin; *AFP* α-fetoprotein; *LDH* lactate dehydrogenase; *IGCCCG* International Germ Cell Consensus Classification Group | | |
