# Supplementary figures and images for: Increased levels of XPA might be the basis of cisplatin resistance in germ cell tumours
Source: BMC Cancer. 2020 Jan 6;20:17. doi: 10.1186/s12885-019-6496-1 (PMC6945513; doi:10.1186/s12885-019-6496-1)

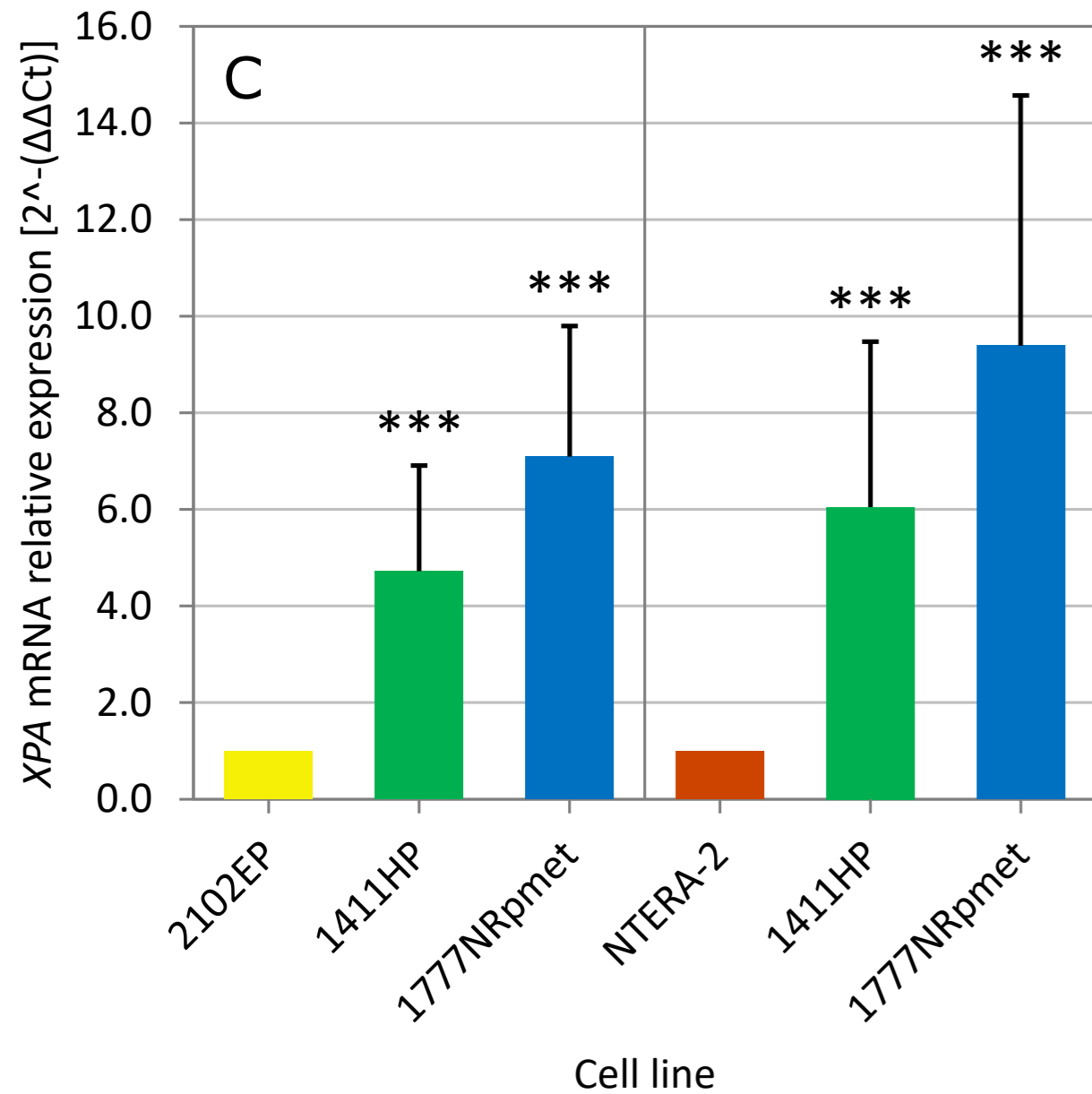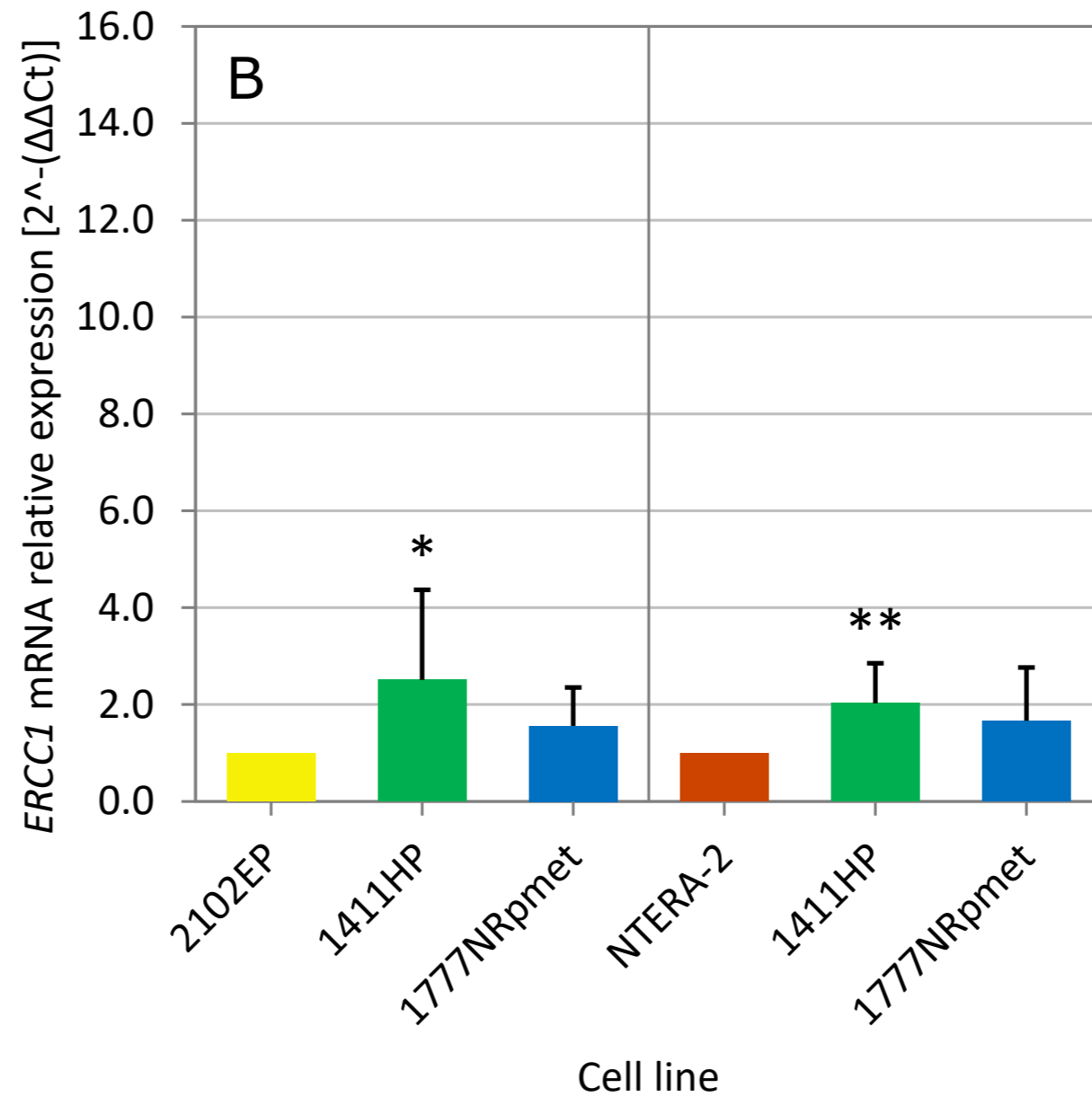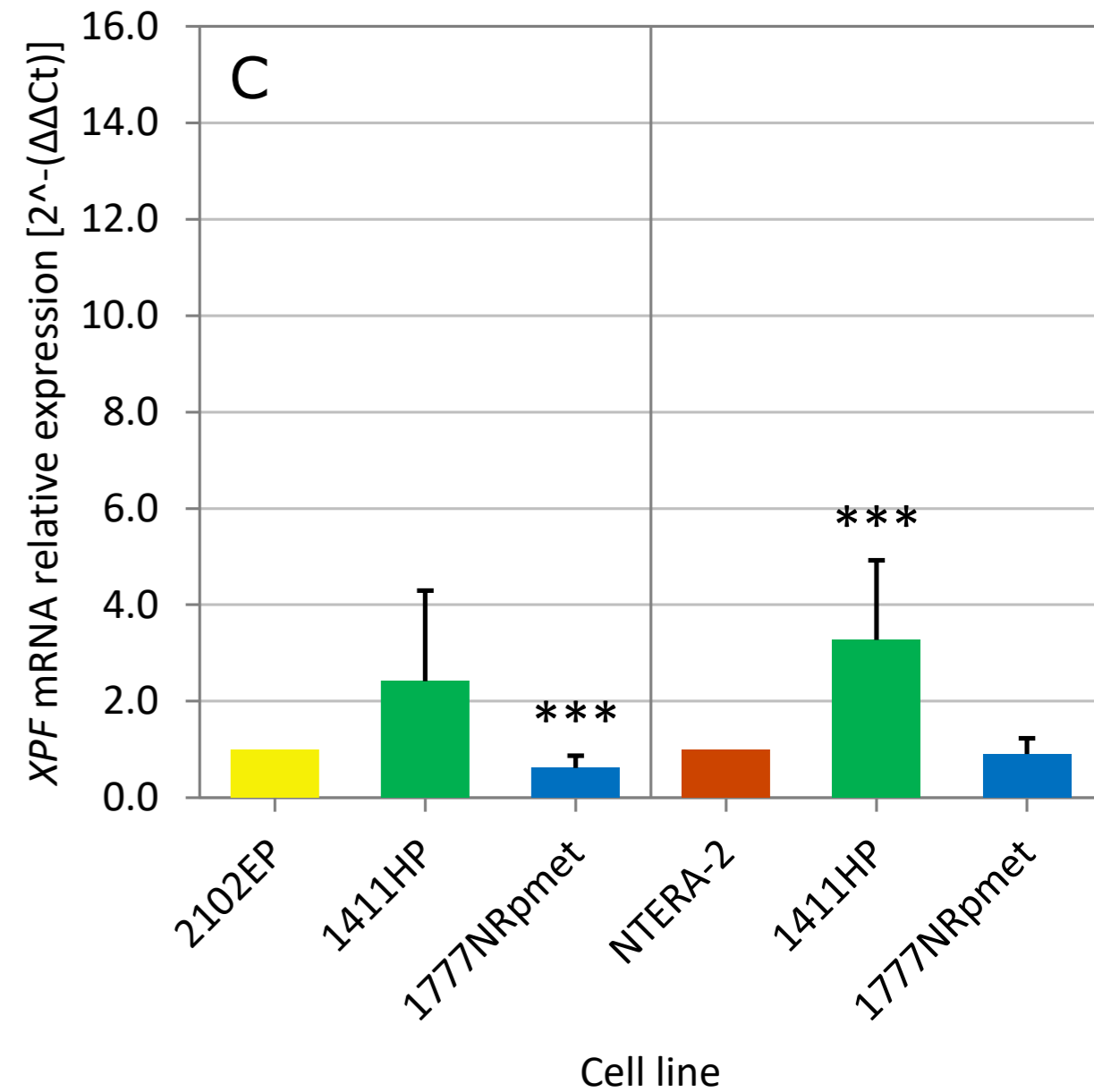

Supplement: Supplementary file 3 — Additional file 3: Figure S1. Comparison of the (a) XPA, (b) ERCC1 and (c) XPF mRNA expression between CDDP-resistant (1411HP and 1777NRpmet) and -sensitive (2102EP and NTERA-2) GCT cell lines. Error bars represent the standard deviation of three technical replicates of three biological replicates. * p ≤ 0.01, ** p ≤ 0.005, *** p ≤ 0.001 [file 12885_2019_6496_MOESM3_ESM.pdf]

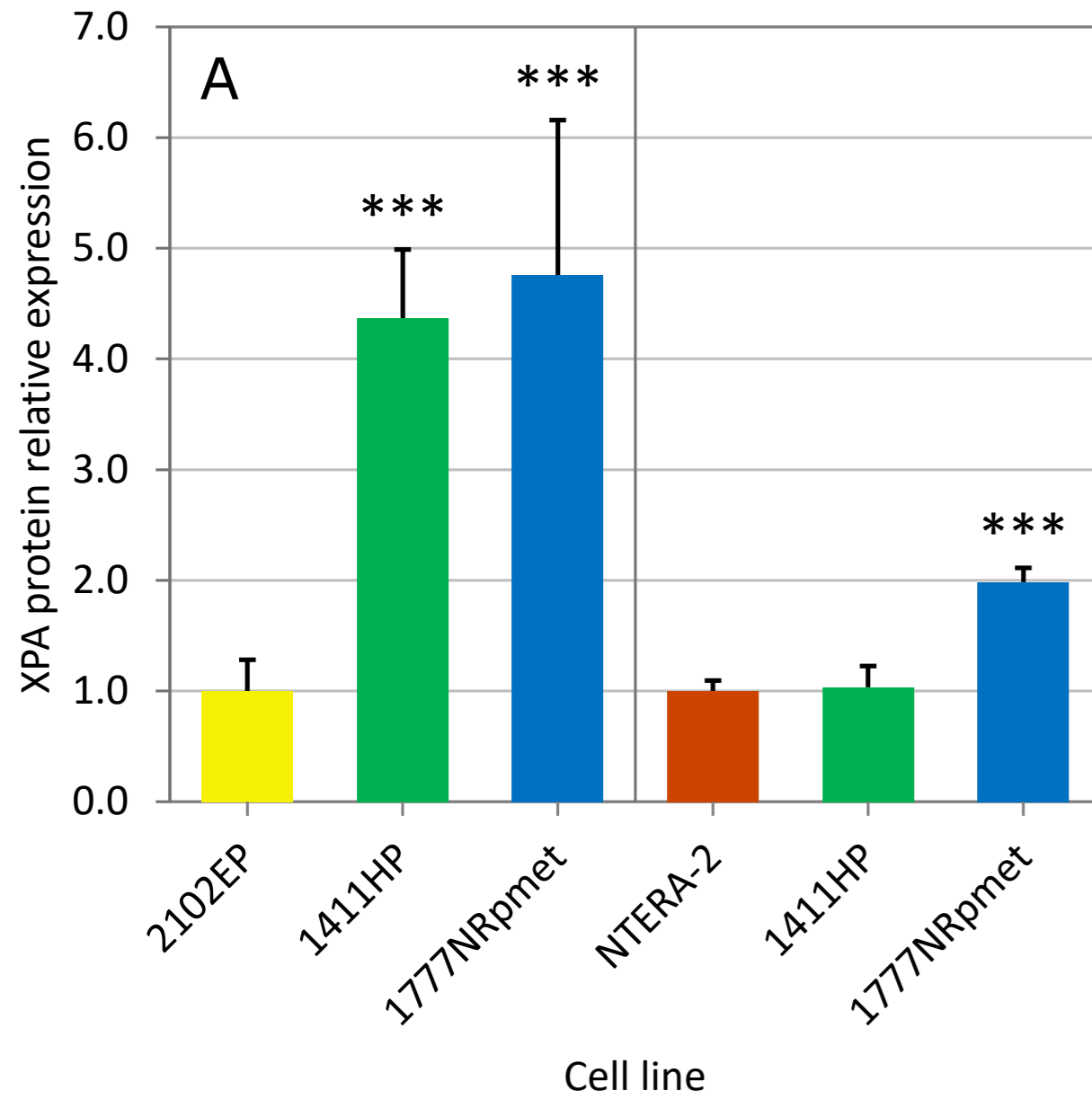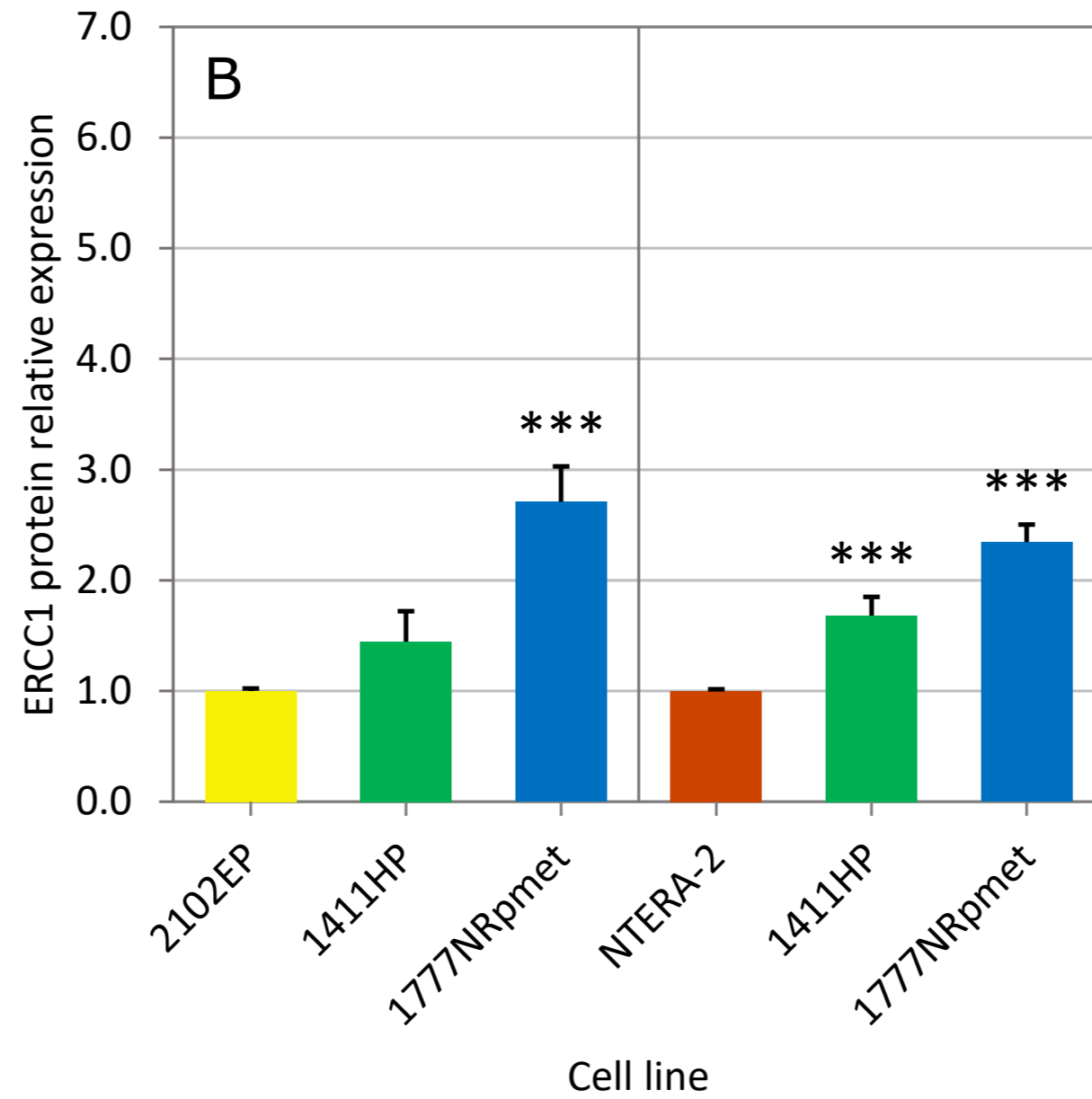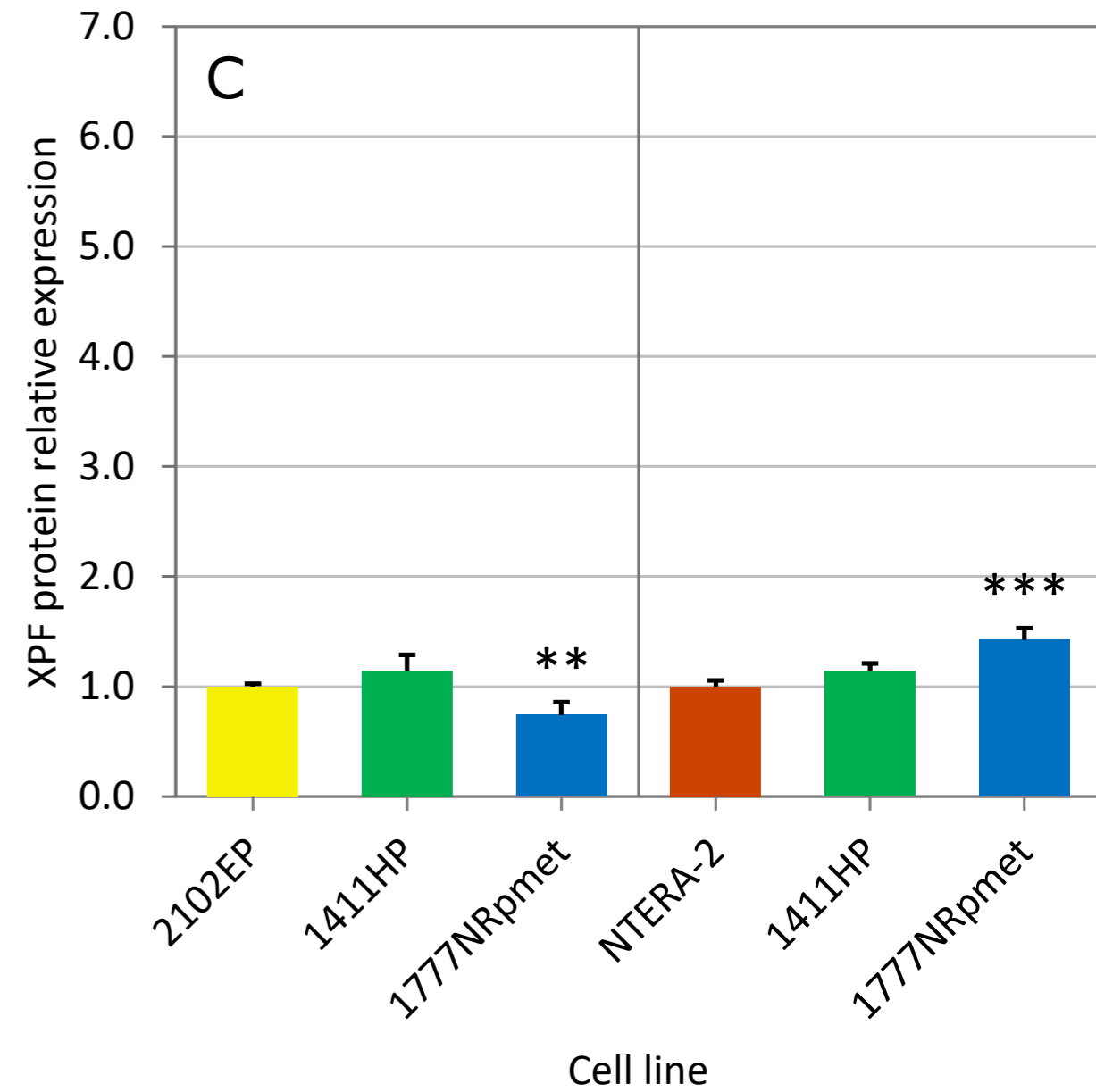

Supplement: Supplementary file 4 — Additional file 4: Figure S2. Comparison of the (a) XPA, (b) ERCC1 and (c) XPF protein expression between CDDP-resistant (1411HP and 1777NRpmet) and -sensitive (2102EP and NTERA-2) GCT cell lines. Upper panels show representative Western blots. Lower panels (graphs) are their quantitative analyses with error bars representing the standard deviation of three technical replicates of three biological replicates. * p ≤ 0.01, ** p ≤ 0.005, *** p ≤ 0.001 [file 12885_2019_6496_MOESM4_ESM.pdf]
